# Comparison of bioavailability and antiplatelet action of ticagrelor in patients with ST-elevation myocardial infarction and non-ST-elevation myocardial infarction: A prospective, observational, single-centre study

**DOI:** 10.1371/journal.pone.0186013

**Published:** 2017-10-12

**Authors:** Piotr Adamski, Joanna Sikora, Ewa Laskowska, Katarzyna Buszko, Małgorzata Ostrowska, Julia M. Umińska, Adam Sikora, Natalia Skibińska, Przemysław Sobczak, Urszula Adamska, Danuta Rość, Aldona Kubica, Przemysław Paciorek, Michał P. Marszałł, Eliano P. Navarese, Diana A. Gorog, Jacek Kubica

**Affiliations:** 1 Department of Principles of Clinical Medicine, Collegium Medicum, Nicolaus Copernicus University, Bydgoszcz, Poland; 2 Department of Pharmacology and Therapy, Collegium Medicum, Nicolaus Copernicus University, Bydgoszcz, Poland; 3 Department of Theoretical Foundations of Biomedical Science and Medical Informatics, Collegium Medicum, Nicolaus Copernicus University, Bydgoszcz, Poland; 4 Collegium Medicum, Nicolaus Copernicus University, Bydgoszcz, Poland; 5 Department of Medicinal Chemistry, Collegium Medicum, Nicolaus Copernicus University, Bydgoszcz, Poland; 6 Department of Cardiology and Internal Medicine, Collegium Medicum, Nicolaus Copernicus University, Bydgoszcz, Poland; 7 Chair of Dermatology, Sexually Transmitted Diseases and Immunodermatology, Faculty of Medicine, Nicolaus Copernicus University in Toruń, Poland; 8 Department of Pathophysiology, Collegium Medicum, Nicolaus Copernicus University, Bydgoszcz, Poland; 9 Department of Health Promotion, Collegium Medicum, Nicolaus Copernicus University, Bydgoszcz, Poland; 10 National Heart & Lung Institute, Imperial College, London, United Kingdom; Medizinische Hochschule Hannover, GERMANY

## Abstract

**Background:**

Data from available studies suggest that the presence of ST-elevation myocardial infarction (STEMI) may be associated with delayed and attenuated ticagrelor bioavailability and effect compared with non-ST-elevation myocardial infarction (NSTEMI).

**Methods:**

In a single-center, prospective, observational trial 73 patients with myocardial infarction (STEMI n = 49, NSTEMI n = 24) underwent a pharmacokinetic and pharmacodynamic assessment after a 180 mg ticagrelor loading dose (LD). Ticagrelor and its active metabolite (AR-C124910XX) plasma concentrations were determined with liquid chromatography tandem mass spectrometry, and their antiplatelet effect was measured with the VASP assay and multiple electrode aggregometry.

**Results:**

During the first six hours after ticagrelor LD, STEMI patients had 38% and 34% lower plasma concentration of ticagrelor and AR-C124910XX, respectively, than NSTEMI (ticagrelor AUC_(0–6)_: 2491 [344–5587] vs. 3991 [1406–9284] ng*h/mL; p = 0.038; AR-C124910XX AUC_(0–6)_: 473 [0–924] vs. 712 [346–1616] ng*h/mL; p = 0.027). STEMI patients also required more time to achieve maximal concentration of ticagrelor (t_max_: 4.0 [3.0–12.0] vs. 2.5 [2.0–6.0] h; p = 0.012). Impaired bioavailability of ticagrelor and AR-C124910XX seen in STEMI subjects was associated with diminished platelet inhibition in this group, which was most pronounced during the initial hours of treatment.

**Conclusions:**

Plasma concentrations of ticagrelor and AR-C124910XX during the first hours after ticagrelor LD were one third lower in STEMI than in NSTEMI patients. This reduced and delayed ticagrelor bioavailability was associated with weaker antiplatelet effect in STEMI.

**Clinical trial registration:**

ClinicalTrials.gov identifier: NCT02602444 (November 09, 2015)

## Introduction

Patients suffering from acute myocardial infarction (AMI) constitute a heterogeneous population and acute treatment is mainly determined by clinical and electrocardiographic features. The presence or absence of ST segment elevation on the ECG largely determines the type and time frame of treatment strategy. Patients with ST-elevation myocardial infarction (STEMI) require immediate revascularization, preferably with primary percutaneous coronary intervention (PCI), while in non-ST-elevation myocardial infarction (NSTEMI) the necessity for invasive treatment depends on individual risk stratification [1, 2].

Dual antiplatelet therapy, with aspirin and one of the P2Y12 receptor antagonists, is the cornerstone of pharmacological treatment in both conservatively and invasively managed AMI patients [1, 2]. The importance of dual antiplatelet therapy stems from the need to reduce the excessive platelet activation and aggregation that is one of the main pathomechanisms of AMI. High platelet reactivity (HPR) on P2Y12 receptor inhibitors is a recognized risk factor for stent thrombosis and can be associated with increased mortality [3]. Thus, patients undergoing PCI for AMI require rapid and potent platelet inhibition.

Ticagrelor, which is an oral, potent, direct and reversibly-binding agent, is the standard of care P2Y12 receptor inhibitor in the setting of AMI [1, 2, 4, 5]. Ticagrelor has linear pharmacokinetics in healthy volunteers and in patients with stable coronary artery disease [6]. Ticagrelor has one major active metabolite, AR-C124910XX, which expeditiously appears in the blood plasma and reaches approximately 30–40% concentration of the main compound [7]. AR-C124910XX exerts an antiplatelet effect comparable to that seen with the parent drug.

However, even novel and powerful P2Y12 receptor inhibitors, like prasugrel or ticagrelor, fail to achieve adequate platelet inhibition in all AMI patients in the first few hours following the loading dose (LD) [8–10]. Of note, the antiplatelet effect of ticagrelor corresponds to its plasma concentration [11]. This indicates that in case of impaired ticagrelor bioavailability, AMI patients may be at risk of inadequate platelet inhibition at a time when it is most desired. Data from available pharmacokinetic/pharmacodynamic (PK/PD) studies suggest that STEMI diagnosis may be associated with delayed and attenuated ticagrelor plasma concentration and action, when compared with NSTEMI patients [10, 12]. Moreover, this adverse relationship can be further aggravated by the administration of morphine, commonly administered in AMI, especially in STEMI [8, 10, 13]. The PK/PD profile of ticagrelor has never been prospectively and directly compared between STEMI and NSTEMI subjects.

In this study, we aimed to compare the PK/PD profiles of ticagrelor and its active metabolite in STEMI and NSTEMI patients.

## Methods

### Study design

The comparison of ticagrelor exposure and effects in STEMI and NSTEMI patients (PINPOINT) study was a phase IV, single-centre, investigator-initiated, prospective, observational trial aimed to compare PK/PD of ticagrelor in patients with STEMI and NSTEMI. The study was conducted in accordance with the principles contained in the Declaration of Helsinki and Good Clinical Practice guidelines. The study was approved by The Ethics Committee of Nicolaus Copernicus University in Toruń, Collegium Medicum in Bydgoszcz, Poland (KB 617/2015). Written informed consent was obtained from each patient prior to any study specific procedures. Key inclusion criteria were: provision of informed consent for angiography and PCI, and diagnosis of STEMI or NSTEMI according to the Third Universal Definition of Myocardial Infarction [14]. Main exclusion criteria were: contraindication to ticagrelor, treatment with any P2Y12 receptor inhibitor within 14 days prior to study enrolment, ongoing treatment with oral anticoagulant or chronic therapy with low molecular weight heparin, active bleeding, Killip class III or IV during screening for eligibility and respiratory failure. The complete list of exclusion criteria has been previously published [15].

Consecutive STEMI and NSTEMI patients admitted to the study site (Cardiology Department, Dr. A. Jurasz University Hospital, Bydgoszcz, Poland) were screened for eligibility. After admission to the study centre, confirmation of the initial diagnosis of STEMI or NSTEMI, and provision of a written informed consent to participate in the trial, all study participants received an oral 300 mg LD of plain aspirin (Polpharma SA, Starogard Gdański, Poland) and 180 mg LD ticagrelor in integral tablets with 250 mL tap water. Subsequently, within 15 minutes of ticagrelor LD, all patients underwent coronary angiography followed by PCI as required. During the periprocedural period, all study participants received unfractionated heparin in body weight adjusted dose according to the European Society of Cardiology (ESC) recommendations [1, 2]. Plasma concentrations of ticagrelor and its active metabolite, as well as their antiplatelet effect, were evaluated according to the sampling schedule employed at our centre in previous PK/PD study (pre-treatment, 0.5 h, 1 h, 2 h, 3 h, 4 h, 6 h and 12 h post ticagrelor LD) [16].

### Endpoints

The primary endpoint of the study was the area under the plasma concentration-time curve for ticagrelor during the first 6 h after administration of ticagrelor LD (AUC_(0–6)_). Secondary endpoints included AUC_(0–6)_ for AR-C124910XX, AUC_(0–12)_ for ticagrelor and AR-C124910XX, maximum concentration (C_max_) of ticagrelor and AR-C124910XX, time to maximum concentration (t_max_) for ticagrelor and AR-C124910XX, platelet reactivity assessed by the vasodilator-stimulated phosphoprotein (VASP) assay and the Multiplate analyzer, percentage of patients with HPR after ticagrelor LD assessed with VASP and Multiplate, time to reach platelet reactivity below the cut-off value for HPR evaluated with VASP and Multiplate.

### Blood sampling

Blood samples for PK/PD evaluation were obtained from an 18-gauge cannula inserted into one of the forearm veins. The first 5 mL blood was discarded to avoid spontaneous platelet activation. In each patient specimens were collected at the eight pre-defined time. Detailed description of blood sample processing has been published in the study protocol (S1 File) [15].

### Pharmacokinetic assessment

Ticagrelor and AR-C124910XX plasma concentrations were analyzed with liquid chromatography coupled with tandem mass spectrometry, using a validated method [15]. Lower limits of quantification were 4.69 ng/mL for both ticagrelor and AR-C124910XX.

### Pharmacodynamic assessment

Platelet reactivity was assessed using VASP and Multiplate. The VASP assay (Biocytex, Inc., Marseille, France) was used in all study participants, while Multiplate (Roche Diagnostics International Ltd., Rotkreuz, Switzerland) was used in all except in those treated with glycoprotein (GP) IIb/IIIa receptor inhibitors. Pharmacodynamic evaluation was performed according to the manufacturers' instructions [17]. HPR was defined as platelet reactivity index >50% for the VASP assay and AUC >46 units (U) for Multiplate [18].

### Sample size calculation

Since there was no reference study comparing the pharmacokinetics of ticagrelor in the acute phase of STEMI and NSTEMI, we decided to perform an internal pilot study. Based on the results obtained from the analysis of the first 45 consecutively enrolled patients (15 with NSTEMI and 30 with STEMI), and assuming a two-sided alpha value of 0.05, we calculated, using the t-test for independent variables, that enrolment of at least 23 patients in each study arm would provide 95% power to demonstrate a significant difference in AUC_(0–6)_ for ticagrelor between patients with different types of AMI as presented in the study protocol (S1 File) [15].

### Statistical analyses

Statistical calculations were performed using the Statistica 12.5 package (StatSoft, Tulsa, OK, USA). Pharmacokinetic calculations and plots were made using the Matlab R2014 software (Mathworks, Natick, MA, USA); trapezoidal rule was applied to calculate AUC. Data for pharmacokinetic features of ticagrelor and AR-C124910XX, pharmacodynamic outcome variables, creatinine, glomerular filtration rate and left ventricle ejection fraction were presented as medians and interquartile ranges. Data for heart rate, systolic and diastolic blood pressure, age and body mass index were presented as means with standard deviations. Both C_max_ and t_max_ were evaluated for the period from 0 to 12 hours. Continuous variables were compared between both study groups with the Student’s t-test and Mann-Whitney U test, depending on the presence or absence of the normal distribution (as assessed by the Shapiro-Wilk test). Comparisons between categorical variables were performed by the chi-square test, with Yates's correction if necessary, or using Fisher's exact test.

## Results

### Baseline characteristics and events within the observation period

Between November 2015 and January 2017, a total of 73 consecutive AMI patients (49 STEMI and 24 NSTEMI) were enrolled. The recruitment continued until the minimum required number of patients were enrolled in the less numerous group (NSTEMI). There were no differences in the baseline characteristics between the two groups, except higher mean left ventricle ejection fraction seen in NSTEMI patients at discharge (Table 1). Of note, as expected numerically greater proportion of STEMI patients were treated with morphine, but this difference was statistically insignificant. Adverse events during the observation period (12 hours after ticagrelor LD) were sporadic and mild (STEMI vs. NSTEMI: Thrombolysis in Myocardial Infarction minimal bleeding 6.1% vs. 8.3%, p = 0.92; dyspnea 2.0% vs. 4.2%, p = 0.83; vomiting 4.1% vs. 4.2%, p = 0.56).

**Table 1:** Baseline characteristics of study participants.

	STEMI n = 49	NSTEMI n = 24	p value
Age in years	62.2 ± 9.4	64.7 ± 9.9	0.24
Age ≥ 70	11 (23)	8 (33)	0.47
Female	11 (23)	9 (38)	0.17
BMI in kg/m2	27.4 ± 4.2	27.4 ± 3.6	0.8
Hypertension	21 (43)	16 (67)	0.056
Diabetes mellitus	7 (14)	3 (13)	0.87
Dyslipidemia	46 (94)	21 (88)	0.63
Current smoker	25 (51)	8 (33)	0.15
Prior MI	5 (10)	2 (8)	0.86
Prior PCI	4 (8)	3 (13)	0.86
Prior CABG	0 (0)	0 (0)	n/a
Congestive heart failure	0 (0)	0 (0)	n/a
Nonhemorrhagic stroke	1 (2)	0 (0)	0.71
Peripheral arterial disease	2 (4)	2 (8)	0.71
Chronic renal disease	0 (0)	0 (0)	n/a
Chronic obstructive pulmonary disease	0 (0)	0 (0)	n/a
Gout	1 (2)	1 (4)	0.8
Heart rate at admission in bpm	78 ± 16	75 ± 14	0.38
SBP at admission in mmHg	137 ± 36	145 ± 20	0.16
DBP at admission in mmHg	82 ± 15	83 ± 15	0.96
Killip class I at admission	42 (86)	22 (92)	0.71
Creatinine at admission in mg/dL	0.85 [0.77–0.98]	0.80 [0.71–0.92]	0.078
GFR at admission in mL/minute	81 [72–96]	85 [78–90]	0.57
Morphine administration	27 (55)	10 (42)	0.69
GP IIb/IIIa inhibitors administration	16 (33)	3 (13)	0.067
Three-vessel coronary artery disease	13 (27)	7 (29)	0.97
LVEF at discharge in %	45 [40–50]	50 [45–51]	0.027

Data are shown as mean ± standard deviation, median [interquartile range] or number (%). BMI: body mass index, bpm: beats per minute; CABG: coronary artery bypass grafting, DBP: diastolic blood pressure, GFR: glomerular filtration rate, GP: glycoprotein, LVEF: left ventricle ejection fraction, MI: myocardial infarction, n/a: not applicable, NSTEMI: non-ST-elevation myocardial infarction, PCI: percutaneous coronary intervention, SBP: systolic blood pressure, STEMI: ST-elevation myocardial infarction.

### Pharmacokinetics

**Fig 1:**
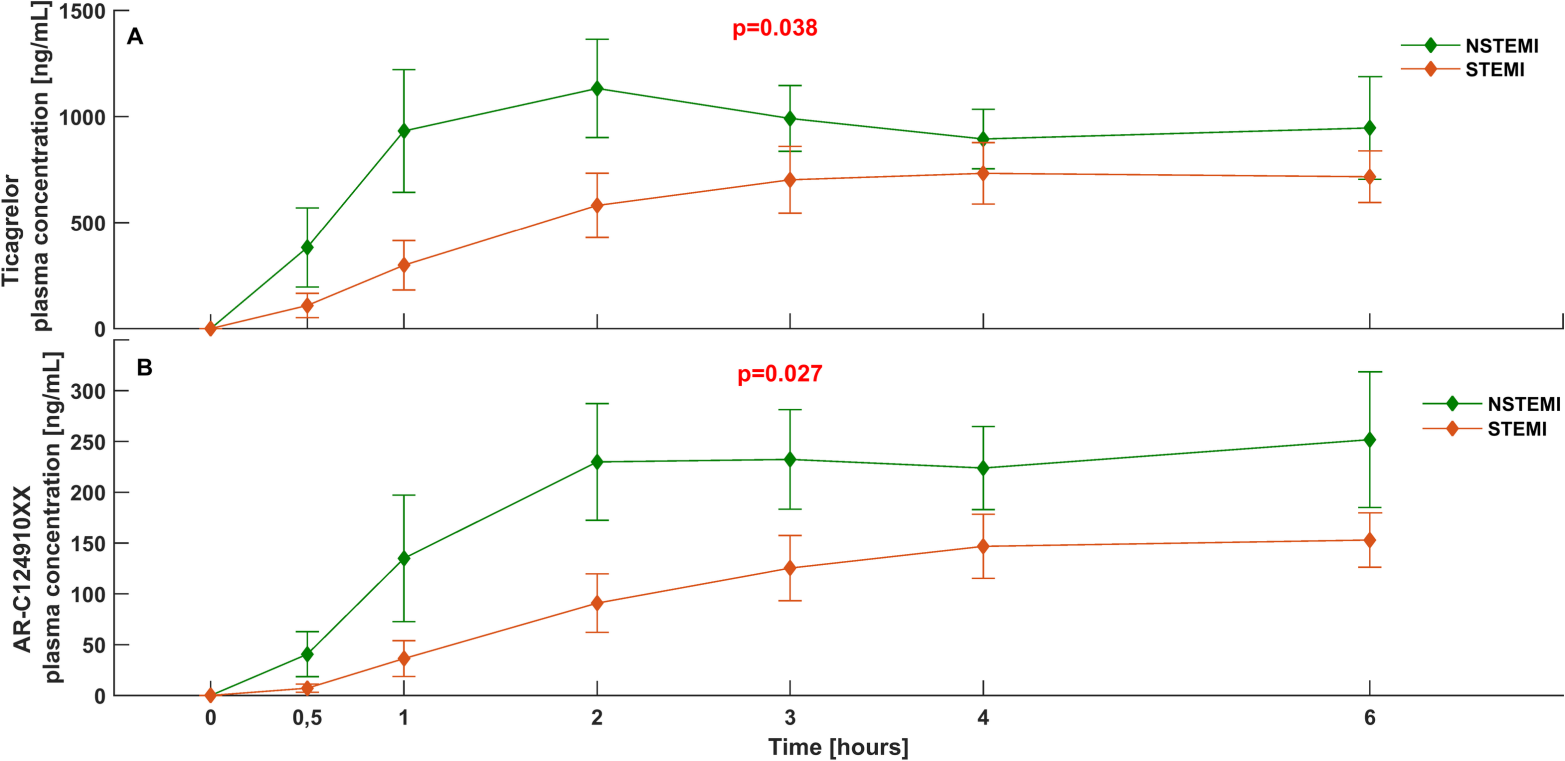
Bioavailability of ticagrelor and AR-C124910XX over time in patients with STEMI and NSTEMI. Plasma concentrations of (A) ticagrelor and (B) AR-C124910XX during the first 6 h after oral administration of a 180 mg ticagrelor loading dose in patients with STEMI and NSTEMI. NSTEMI: non-ST-elevation myocardial infarction, STEMI: ST-elevation myocardial infarction.

The total ticagrelor bioavailability during the first 6 h after the LD, as measured by the AUC_(0–6)_, was 38% lower in STEMI than in NSTEMI patients (2491 [344–5587] vs. 3991 [1406–9284] ng*h/mL; p = 0.038) (Fig 1A). Similarly, a 34% lower AUC_(0–6)_ for AR-C124910XX was observed in STEMI group (473 [0–924] vs. 712 [346–1616] ng*h/mL; p = 0.027) (Fig 1B). At 12 h post LD, NSTEMI patients still had numerically higher total exposure to ticagrelor and its active metabolite, but these differences diminished over time and were no longer significant (AUC_(0–12)_ for ticagrelor in STEMI vs. NSTEMI: 5676 [3441–10955] vs. 8597 [4084–14127] ng*h/mL; p = 0.27; AUC_(0–12)_ for AR-C124910XX in STEMI vs. NSTEMI: 1383 [766–2182] vs. 1523 [1005–2867] ng*h/mL; p = 0.30). Moreover, STEMI patients required longer time to achieve C_max_ of ticagrelor (t_max_: 4.0 [3.0–12.0] vs. 2.5 [2.0–6.0] h; p = 0.012), however neither C_max_ of ticagrelor (STEMI vs. NSTEMI: 1107 [652–1741] vs. 1161 [605–2545] ng*h/mL; p = 0.31) nor AR-C124910XX (STEMI vs. NSTEMI: 228 [140–346] vs. 191 [137–437] ng*h/mL; p = 0.72) differed between the study groups. In a post hoc analysis, none of the prespecified PK endpoints differed between patients with STEMI treated with morphine versus opioid-naive patients with STEMI. Likewise, when patients with NSTEMI were compared based on the administration of morphine, no significant differences in the main PK features were observed, with the exception of delayed t_max_ in opioid-treated patients (1.0 [2.0–2.0] vs. 4.0 [3.0–6.0] h; p = 0.032).

### Platelet reactivity

**Fig 2:**
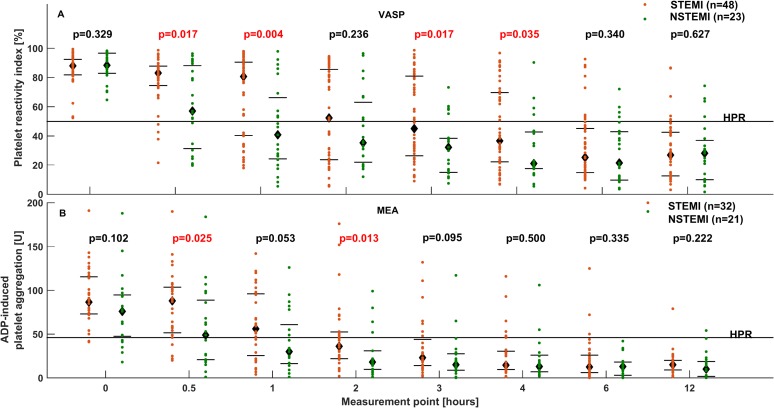
Platelet reactivity over time in STEMI and NSTEMI patients. Platelet reactivity evaluated with (A) the VASP assay and (B) Multiplate at baseline, and at 0.5 h, 1 h, 2 h, 3 h, 4 h, 6 h, and 12 h after administration of a 180 mg ticagrelor loading dose in patients with STEMI and NSTEMI. NSTEMI: non-ST-elevation myocardial infarction, STEMI: ST-elevation myocardial infarction, VASP: vasodilator-stimulated phosphoprotein.

**Fig 3:**
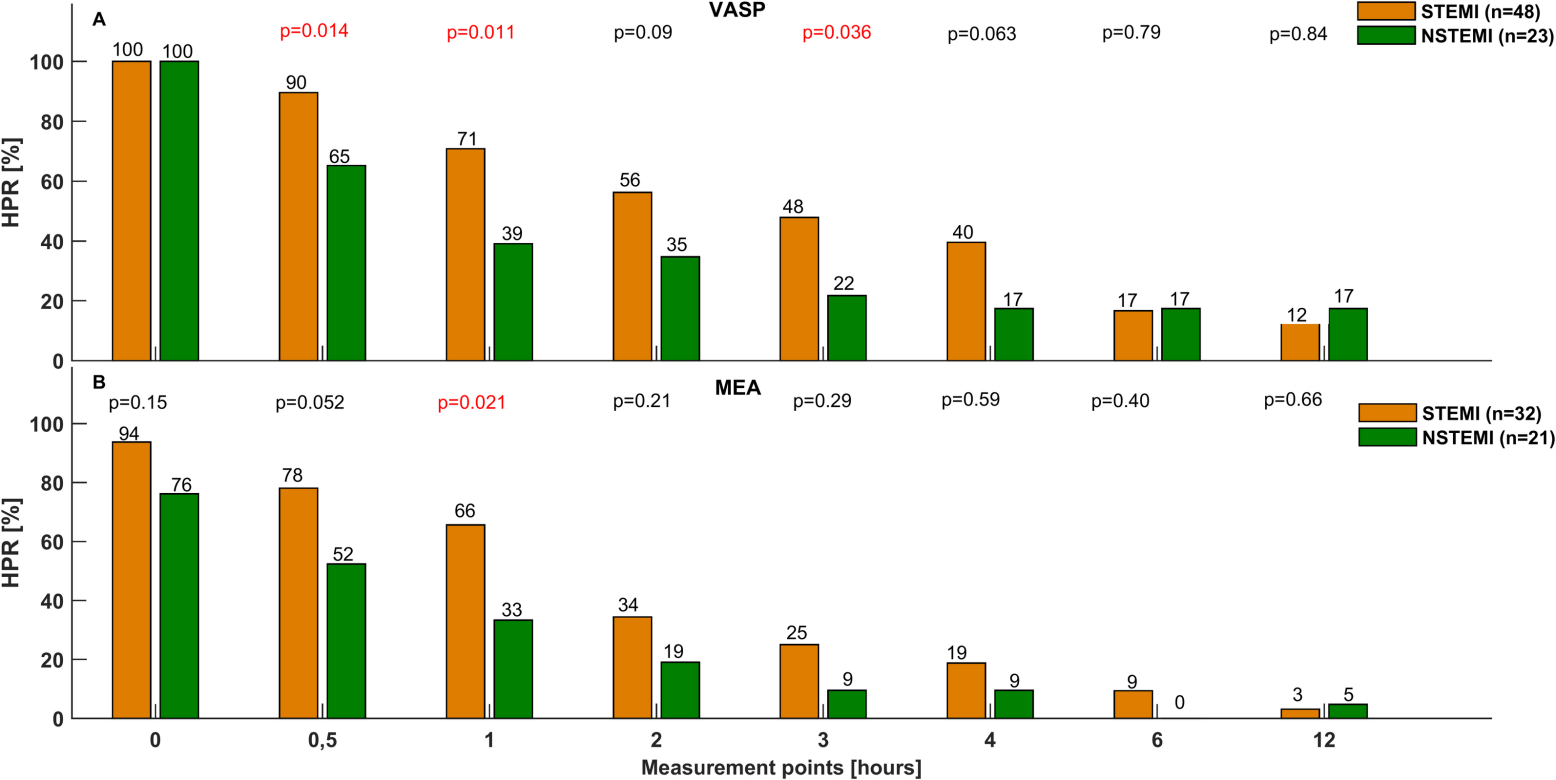
Prevalence of high platelet reactivity over time in STEMI and NSTEMI patients. Proportion of patients with high platelet reactivity assessed with (A) the VASP assay and Multiplate (B) at baseline, and at 0.5 h, 1 h, 2 h, 3 h, 4 h, 6 h, and 12 h after administration of a 180 mg ticagrelor loading dose in patients with STEMI and NSTEMI. HPR: high platelet reactivity, NSTEMI: non-ST-elevation myocardial infarction, STEMI: ST-elevation myocardial infarction, VASP: vasodilator-stimulated phosphoprotein.

The impaired bioavailability of ticagrelor in STEMI patients was reflected by reduced platelet inhibition, compared with NSTEMI patients. These differences were seen with both the VASP assay and Multiplate, and were most pronounced during the initial hours after ticagrelor LD. Platelet reactivity was significantly higher in STEMI than in NSTEMI patients at 0.5 h, 1 h, 3 h and 4 h when evaluated with VASP (0.5 h: 83.1 [74.5–87.8] vs. 57.0 [30.2–89.4] %; p 0.017; 1 h: 80.7 [40.4–90.5] vs. 40.8 [23.7–69.2] %; p = 0.004; 3 h: 45.0 [26.4–80.9] vs. 32.2 [14.7–38.7] %; p = 0.017; 4 h: 36.5 [22.2–69.7] vs. 21.1 [17.5–43.1] %; p = 0.035; Fig 2A) and at 0.5 h and 2 h when assessed with Multiplate (0.5 h: 88.0 [51.5–103.5] vs. 49.0 [21.0–86.0] U; p = 0.025; 2 h: 36.0 [22.0–52.5] vs. 18.0 [10.0–31.0] U; p = 0.013; Fig 2B). In addition, it took longer in STEMI than in NSTEMI patients to achieve platelet inhibition below the cut-off value for HPR for both VASP (2.5 [1.0–6.0] vs. 1.0 [0.5–2.0] h; p = 0.004) and Multiplate (2.0 [0.5–3.0] vs. 0.5 [0.5–2.0] h; p = 0.029), evidenced by greater prevalence of HPR during the first hours after ticagrelor LD (Fig 3).

## Discussion

To our knowledge, the PINPOINT study is the first trial to prospectively demonstrate differences in ticagrelor PK/PD between STEMI and NSTEMI patients. Presence of STEMI was associated with lower concentrations of ticagrelor and AR-C124910XX during the first six hours after ticagrelor LD. Moreover, time to reach maximal plasma concentration of ticagrelor was longer in STEMI patients. The reduced bioavailability of ticagrelor observed in this group translated into a weaker antiplatelet effect, which was shown consistently with two different tests of platelet function. Importantly, differences in ticagrelor PK/PD between STEMI and NSTEMI were most pronounced in the initial phase after the LD and attenuated over time.

Recent data show that even though ticagrelor is characterized by swift and potent antiplatelet action, a considerable number of AMI patients may still suffer from inadequate platelet inhibition during the first hours of treatment [8–10]. Gender, age, concomitant food intake, preloading with clopidogrel or genetic effects have been reported not to affect ticagrelor absorption or its antiplatelet effect [11, 19–21]. On the other hand, AMI patients receiving morphine are at greater risk of attenuated ticagrelor bioavailability and inadequate platelet inhibition [10]. Multiple regression analysis of the IMPRESSION study revealed that opioid treatment is not the only predictor of lower ticagrelor concentration in AMI patients—in the trial population (STEMI and NSTEMI patients) the presence of STEMI independently correlated with reduced ticagrelor concentration (R^2^ = 0.17; beta-coefficient = -0.28; p = 0.014) [10]. Similar conclusions were drawn from a post hoc subanalysis of the HARMONIC study, where STEMI patients had decreased overall and delayed maximum ticagrelor and AR-C124910XX concentrations when compared with NSTEMI patients, which subsequently led to weaker platelet inhibition in STEMI [12]. Likewise, Franchi et al. reported that STEMI patients suffer from impaired ticagrelor absorption in comparison with healthy subjects and patients with stable coronary artery disease, which was reflected by delayed onset of platelet inhibition and importantly was also observed in opioid-naive STEMI patients [9]. In contrast, NSTEMI patients not receiving morphine have ticagrelor PK/PD profile similar to that of patients with stable coronary artery disease [22].

The mechanism leading to disturbed ticagrelor PK/PD in patients presenting with STEMI is most likely multifactorial and related to impaired intestinal absorption. The severe clinical course of STEMI may lead to selective shunting of blood flow and gastrointestinal tract hypoperfusion through hemodynamic instability, decreased cardiac output, adrenergic activation and peripheral vasoconstriction [23, 24]. The systemic and hemodynamic consequences of NSTEMI are generally less severe, which could explain why NSTEMI patients have ticagrelor PK/PD profile comparable to that observed in stable coronary artery disease [22]. Vomiting, which is often seen in AMI, may further reduce the bioavailability of orally ingested drugs [13]. Morphine, administered more frequently in STEMI than in NSTEMI, can attenuate the availability of ticagrelor further by inducing emesis and decelerating intestinal transit [10, 25, 26].

Delayed absorption of ticagrelor and subsequent reduction of its antiplatelet effect in STEMI can lead to HPR, which increases the risk of adverse thrombotic consequences, especially in the early phase after invasive treatment [3]. Although the clinical significance of our findings is unknown, several methods to improve ticagrelor PK/PD in STEMI can be proposed. Increasing LD regimens would be a rational choice, however this alluring concept has been shown to be ineffective in improving ticagrelor antiplatelet effect in the STEMI setting [9]. On the other hand, crushing of ticagrelor tablets appears to be an economical and efficient method to enhance and accelerate its bioavailability and onset of action in patients with acute coronary syndrome [27–29]. Alternative administration strategies, such as chewed ticagrelor, were also shown to provide superior platelet inhibition compared with integral pills [30, 31]. Moreover, insufficient platelet blockade after ticagrelor LD in STEMI could be overcome by bridging therapy with intravenous agents. In a small, prospective and nonrandomized study cangrelor, the only available parenteral P2Y12 receptor inhibitor, markedly reduced platelet reactivity and basically eliminated HPR phenomenon in STEMI patients during the first hour after ticagrelor LD [32]. In STEMI patients with increased platelet reactivity on prasugrel due to concomitant morphine administration, intravenous administration of the GP IIb/IIIa receptor inhibitor abciximab resulted in immediate and efficient platelet inhibition [33].

In a real-world setting, the PK/PD differences between STEMI and NSTEMI may be even greater than that observed in our trial. NSTEMI patients included in the PINPOINT study comprised mainly high-risk and very-high-risk NSTEMI subjects, who frequently required opiates to alleviate chest pain or dyspnea (patients were classified as very high-risk due to recurrent or ongoing chest pain refractory to medical treatment, and as high-risk due to rise or fall in cardiac troponin compatible with AMI, dynamic ST- or T-wave changes, or GRACE score >140 [1]; average GRACE score was 145.5 ± 31.7 points according to the GRACE 2.0 risk calculator [34]). Intermediate- and low-risk NSTEMI patients, who were a minority in the current study, would likely require less analgesia and are likely to have ticagrelor PK/PD profile resembling that seen in the stable setting.

### Study limitations

Several limitations of our study have to be acknowledged. First, the trial population was insufficient to evaluate clinical endpoints or to perform subgroup analyses. Second, patients receiving morphine were not excluded from the study. As expected, morphine administration rate was numerically higher in STEMI than in NSTEMI group, but this difference was not statistically significant. Third, morphine was used at the discretion of the paramedics or the attending physicians, although they were encouraged to administer a standardized dose of 5 mg intravenously, if required. Moreover, information on the time span between the onset of symptoms and ticagrelor LD was not collected. It cannot be excluded that the obtained results could be partially explained by administration of ticagrelor LD in more acute phase of AMI observed in STEMI due to expected shorter time from the onset of ischemia to ticagrelor LD in this group (e.g. because of delay required to obtain concentration of troponins needed to make the NSTEMI diagnosis). Finally, our study did not aim to explore the exact mechanism responsible for the observed differences.

## Conclusions

The bioavailability of ticagrelor during the first six hours after the LD is over one-third lower in STEMI compared with NSTEMI patients. Subsequently, in the initial hours after ticagrelor LD NSTEMI subjects achieve greater platelet inhibition and require less time to reach platelet reactivity below the cut-off values for HPR than STEMI patients. The clinical significance of these findings warrant further research.

## Supporting information

S1 File The PINPOINT study protocol. (PDF)

S1 Table STROBE checklist for the PINPOINT study. (DOC)
